# Effect of electrode position on azo dye removal in an up-flow hybrid anaerobic digestion reactor with built-in bioelectrochemical system

**DOI:** 10.1038/srep25223

**Published:** 2016-04-28

**Authors:** Min-Hua Cui, Dan Cui, Hyung-Sool Lee, Bin Liang, Ai-Jie Wang, Hao-Yi Cheng

**Affiliations:** 1 State Key Laboratory of Urban Water Resource and Environment, Harbin Institute of Technology, Harbin 150090, PR China; 2 Key Laboratory of Environmental Biotechnology, Research Center for Eco-Environmental Sciences, Chinese Academy of Sciences, Beijing 100085, PR China; 3 Department of Civil and Environmental Engineering, University of Waterloo, 200 University Avenue West Waterloo, Ontario N2L 3G1, Canada

## Abstract

In this study, two modes of hybrid anaerobic digestion (AD) bioreactor with built-in BESs (electrodes installed in liquid phase (R1) and sludge phase (R2)) were tested for identifying the effect of electrodes position on azo dye wastewater treatment. Alizarin yellow R (AYR) was used as a model dye. Decolorization efficiency of R1 was 90.41 ± 6.20% at influent loading rate of 800 g-AYR/ m^3^·d, which was 39% higher than that of R2. The contribution of bioelectrochemical reduction to AYR decolorization (16.23 ± 1.86% for R1 versus 22.24 ± 2.14% for R2) implied that although azo dye was mainly removed in sludge zone, BES further improved the effluent quality, especially for R1 where electrodes were installed in liquid phase. The microbial communities in the electrode biofilms (dominant by *Enterobacter)* and sludge (dominant by *Enterococcus)* were well distinguished in R1, but they were similar in R2. These results suggest that electrodes installed in liquid phase in the anaerobic hybrid system are more efficient than that in sludge phase for azo dye removal, which give great inspirations for the application of AD-BES hybrid process for various refractory wastewaters treatment.

Azo dye is a serious contaminant that often found in industrial wastewaters, such as textile, paper, cosmetic and food industries^1^. Discharge of azo dye wastewaters to water bodies without appropriate treatment could cause serious environmental problems, such as aesthetic problems, deterioration of receiving water quality and restriction in light penetration^2^. Biological treatment processes have been widely used for azo dye removal^2^,^3^, and anaerobic degradation of azo dye seems more attractive than aerobic processes due to the cost effectiveness^4^,^5^. However, relatively low treatment efficiency as well as slow reaction kinetics has restricted the deployment of anaerobic azo dye wastewater treatment in field.

Bioelectrochemical system (BES) possesses a great potential for transforming nitro aromatics, antibiotics, chlorophenol, halogenide and hexavalent chromium into biodegradable and less toxic products^6^,^7^,^8^. Several BES works have been tested for decolorizing azo dyes^9^,^10^,^11^. Abiotic or biotic cathodes in BESs could serve as an efficient electron donor for reduction of azo dyes. With small external power supply, azo dye decolorization efficiency and reduction rate were improved as compared to conventional anaerobic processes^9^. While BES may not replace conventional biological treatment processes, but could integrate with anaerobic treatment as a hybrid system to improve effluent quality. These hybrid systems were tested for azo dye and *p*-nitrophenol removal^12^. For instance, the removal efficiency of azo dye was enhanced by 8.2% (86.9 ± 6.3% versus 95.1 ± 1.5%) after installing BES in an anaerobic baffled reactor^13^. There was, however, no detailed information about the effect of BES position on azo dye removal in the hybrid anaerobic reactors, although these engineering aspects are significant for commercialization of the hybrid systems; especially, BES position between liquid and sludge zones could be critical for azo dye removal, due to microbial competition or electrode clogging issues^14^,^15^,^16^. In addition, the contribution of electrochemical reduction to the overall removal of azo dyes seems unclear.

In this study, an up-flow hybrid anaerobic digestion reactor with built-in BES was developed for azo dye wastewater treatment. With a BES reactor as the control, the roles of BES in the hybrid reactors were evaluated. In addition, the electrode positions in the hybrid process were optimized (in liquid phase or sludge phase), along with analysis of microbial community structures.

## Results and Discussion

### Comparison of decolorization performance

**Figure 1:**
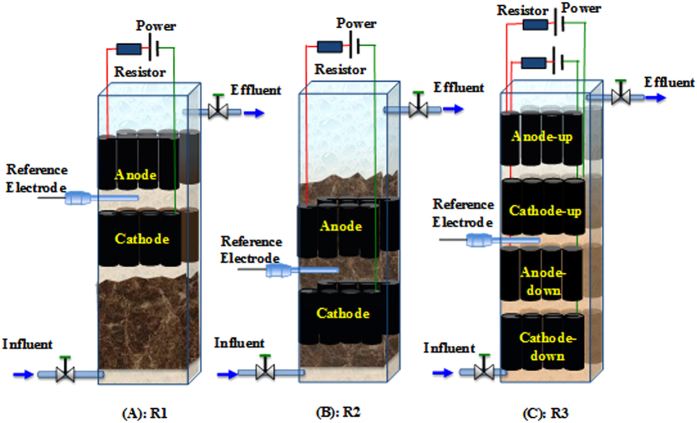
Schematic representation of the three anaerobic hybrid reactors: (**A**) R1, BES installed in liquid phase; (**B**) R2, BES installed in sludge phase; (**C**) R3, BES with two pairs of electrodes.

To evaluate the effect of electrode positions on azo dye removal in the hybrid anaerobic reactors with built-in BES, three reactors equipped with different electrode positions and configurations were compared. The first reactor (**R1 in** Fig. 1A) was filled with anaerobic sludge to a half of the reactor and electrodes were immersed in liquid phase (supernatant). The second reactor (**R2 in** Fig. 1B) was filled with anaerobic sludge to a half of the reactor, and electrodes were placed in sludge zone. The third reactor **(R3 in** Fig. 1C) was equipped with two pairs of electrodes (two bundles of anodes and cathodes), which is a control for assessing the role of sludge zone in R1 and R2. To prevent the possible biotoxicity inhibition of Alizarin Yellow R (AYR) on the anode-respiring microbes, the anodes were installed at the upside of the corresponding cathodes in the three reactors.

**Figure 2:**
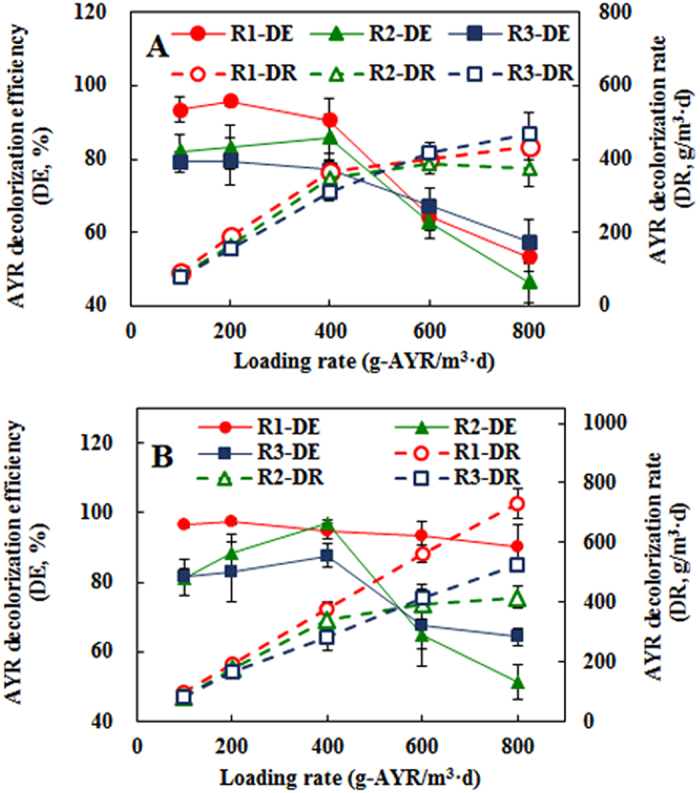
Comparison of AYR decolorization in different reactors: (**A**) AYR decolorization efficiencies and decolorization rates at open circuit; (**B**) AYR decolorization efficiencies and decolorization rates at closed circuit.

At open circuit, the decolorization efficiency (DE) in R1 ranged from 90.78 ± 5.95 to 95.87 ± 1.34% at lower AYR loading rates (ALR) (<400 g AYR/m^3^·d), which fluctuated from 82.22 ± 4.47 to 86.00 ± 6.31% in R2 and from 77.13 ± 4.46 to 79.46 ± 6.52% in R3, respectively (Fig. 2A). When ALR reached to 800 g AYR/m^3^·d, DE sharply dropped to 53.37 ± 4.00% in R1. The same trend was observed in R2 and R3. At closed circuit, DE was improved for all three reactors at ALR < 400 g AYR/m^3^·d (Fig. 2B). Interestingly, DE in R1 showed over 90% even at higher ALR (800 g AYR/ m^3^·d), while DE significantly decreased in R2 and R3 at the higher ALR. This result indicates that electrodes immersing in supernatant (liquid phase) is better than that in sludge zone. When BES was immersed in sludge zone, other microorganisms or particulate matter may seriously inhibit the metabolism of anode-respiring microorganisms. The literature reported that space competition on anodes between anode-respiring microorganisms and non anode-respiring microorganisms became important in BES fed with particulate matter including microorganisms^17^. The growth of non anode-respiring microorganism on anodes would also deteriorate mass transfer of electron donor or acceptor (AYR) in biofilms. As a result, the AYR decolorization rate increased linearly with ALR only in R1 (y = 0.9081x + 11.612, R^2^ = 0.9994, y: AYR decolorization rate, x: ALR) and its maximum rate reached at 730.61 ± 49.59 g AYR/m^3^·d (Fig. 2B). High DE in open-circuit R1 indicates significant AYR reduction by fermenters or methanogens in sludge and suspension (non anode-respiring microorganisms and cathodic reduction), but consistent improvement of DE in closed-circuit R1 at high AYR loading rate clearly proves the significance of BES for AYR reduction.

### Products accumulation and COD removal

**Figure 3:**
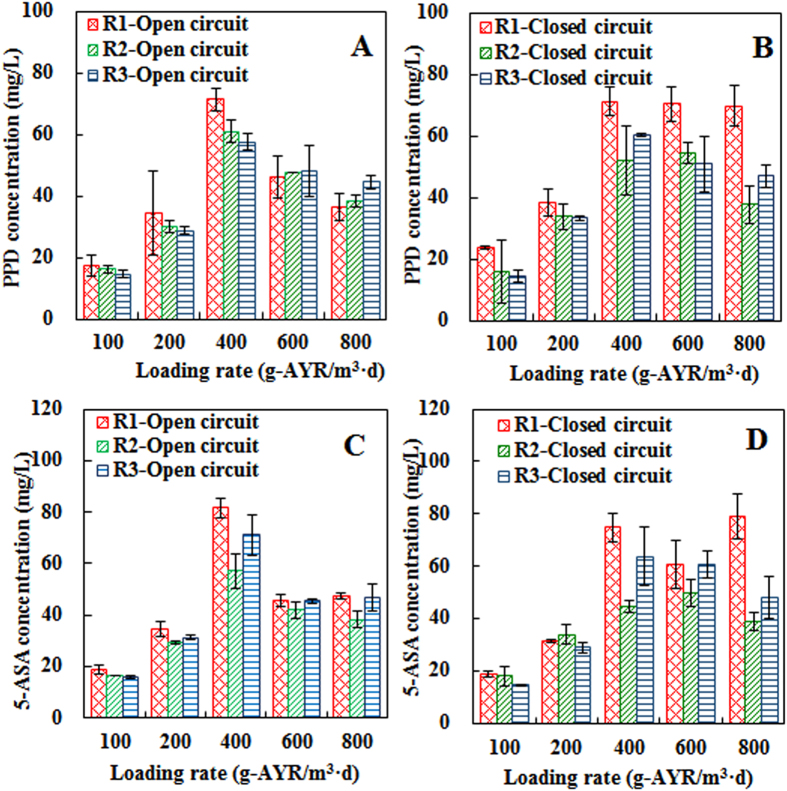
Concentrations of PPD and 5-ASA in different reactors: (**A**) PPD concentration at open circuit; (**B**) PPD concentration at closed circuit; (**C**) 5-ASA concentration at open circuit; (**D**) 5-ASA concentration at closed circuit.

AYR degradation pathway had been proposed by previous literatures: 1 mole AYR reduction can produce 1 mol PPD and 1 mol 5-ASA theoretically^18^,^19^. The formation of these reduced products clearly revealed the removal of AYR was due to the reduction. The formation efficiencies (ratio of measured value and theoretical value) of PPD were over 90% among all three reactors, while the accumulated 5-ASA was less with the formation efficiencies varied from 60% to 85%. This result consists with our previous work and is likely because PPD persist in anaerobic reactor while anaerobic microorganisms, in particular of the methanogenic consortia, could mineralize 5-ASA^19^,^20^. As shown in Fig. 3, PPD and 5-ASA concentrations were highest at 400 g-AYR/m^3^·d under open circuit and there were no distinctive differences among the three reactors. These patterns were consistent to the trend of AYR decolorization. At closed circuit, concentrations of PPD and 5-ASA were simultaneously increased in all reactors at ALR < 400 g/m^3^·d. When ALR increased to 800 g/m^3^·d, PPD and 5-ASA accumulations in R1 kept at 69.64 ± 6.59 and 79.00 ± 8.70 mg/L, but sharply decreased to 37.73 ± 6.19 and 38.66 ± 3.45 mg/L in R2 and 46.89 ± 3.77 and 48.23 ± 7.99 mg/L in R3, respectively. Comparing open circuit modes with closed ones, BES improved the accumulations of two intermediates by 47.40% and 40.11% in R1, indicating more AYR reduction via bioelectrochemical reaction.

**Figure 4:**
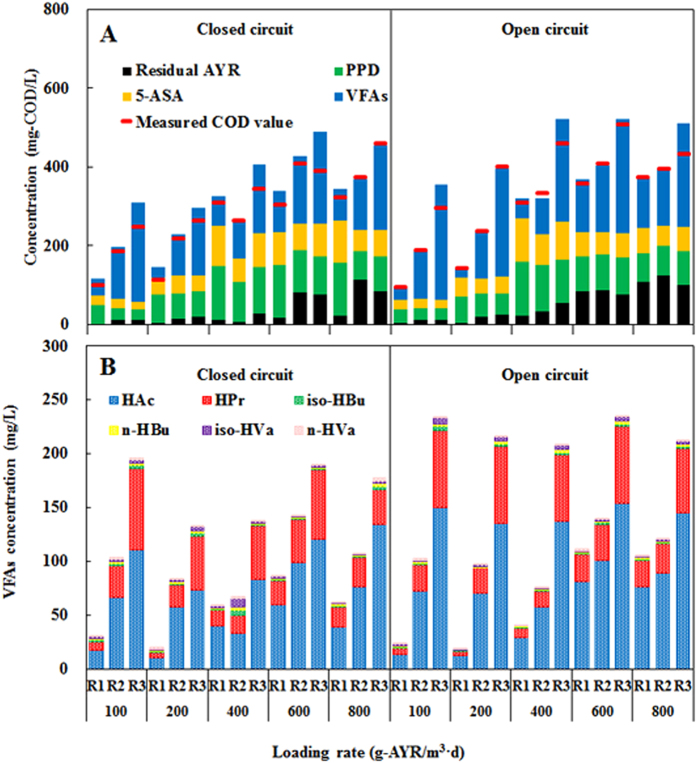
(**A**) COD balance of three reactors at open and closed circuit (AYR converts into COD with coefficient of 1.17; PPD converts into COD with coefficient of 1.93; 5-ASA converts into COD with coefficient of 1.36; VFAs converts into COD with coefficients of 1.07 for HAc, 1.51 for HPr, 1.82 for HBu, 2.04 for HVa.); (**B**) VFAs concentrations in effluents.

COD removal in the three reactors tended to decrease with increasing ALR at both open and closed circuit. R1 presented superior COD removal to R2 and R3. At 100 g-AYR /m^3^ d, R1 showed the COD removal efficiencies of 82.76 and 81.60% at open and closed circuit, respectively, which were nearly 15–37% higher than those in the other two reactors (Fig. 4A). At the highest ALR of 800 g/m^3^ d, COD removal declined to 54.76% for R1 operated at closed circuit, but it was still 7.32% and 19.29% higher than that for R2 and R3, respectively. Poor COD removal in R3 might be due to less biomass on the electrodes. As shown in Fig. 4B, acetate acid (HAc) and propionic acid (HPr) were the main VFAs for all reactors. The other VFAs including iso- butyric acid (0.52–5.37 mg/L), n-butyric acid (0.53–5.41 mg/L), iso-valeric acid (0.67–7.77 mg/L) and n-valeric acid (0.50–3.63 mg/L) were negligible. At ALR of 800 g/m^3^·d, VFAs was 106.21 mg/L for R1 at open circuit, but it significantly decreased to 62.57 mg/L at closed circuit. In comparison, VFAs for R2 and R3 (closed circuit) were relatively higher. Power supply enhanced VFAs removal by 41.09% in R1, indicating efficient VFA utilization by anode-respiring microorganisms. The lower VFAs accumulation and better COD removal in R1 also support the higher metabolic activity of anode-respiring microorganisms when electrodes are placed in the liquid phase.

### Contribution of BES to AYR reduction

**Figure 5:**
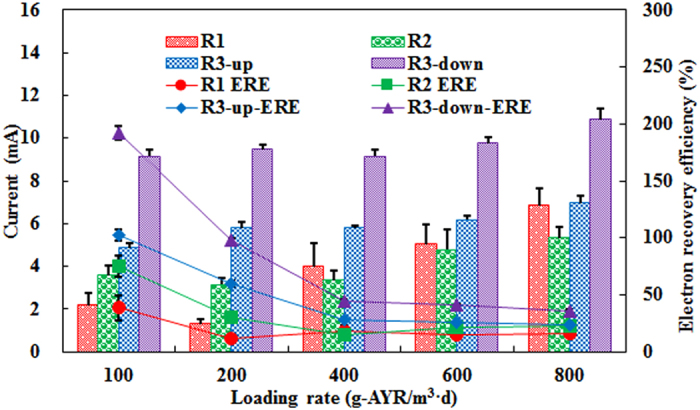
Currents and electron recovery efficiencies (EREs) for different pairs of electrodes.

Currents and electron recovery efficiencies in the hybrid reactors (R1 and R2) were much lower than that in the sole BES (R3), as shown in Fig. 5. At ALR of 800 g/m^3^ d, currents of R1 and R2 were 6.85 ± 0.78 and 5.34 ± 0.51 mA, respectively. Similar current was observed at an upward electrode pair in R3 (6.97 ± 0.31 mA), but a downward electrode pair in R3 generated relatively high current (10.88 ± 0.48 mA). Electron recovery efficiencies for R1 and R2 were 16.23 ± 1.86%, 22.24 ± 2.14% which were 23.23 ± 1.02% (upward electrodes) and 36.27 ± 1.60% (downward electrodes) for R3. It is evident that the total AYR removal was improved in the hybrid system. However, these small electron recovery efficiencies indicate that AYR reduction would mainly occur in sludge zone^13^, suggesting that integration of BES with anaerobic digestion as a hybrid system actually would mitigate BES contribution to AYR removal, including R1.

### Microbial community

**Figure 6:**
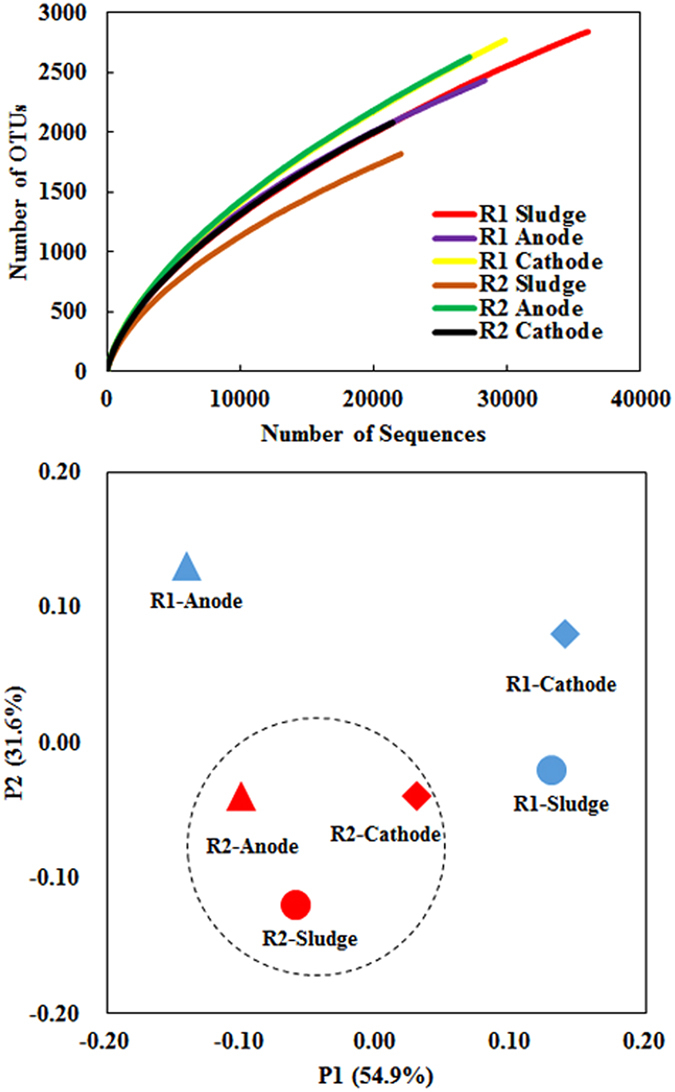
(**A**) Rarefaction curves based on the 16S rRNA gene amplicons sequencing. The OTUs were defined of 3% distances; (**B**) Principal component analysis (PCA) of bacterial communities from the six samples.

Over 20000 16S rRNA gene sequences were identified for each sample by Illumina MiSeq sequencing. As shown in Fig. 6A, operational taxonomic units (OTUs) at 3% distance were 2841, 2434 and 2771 for sludge, anode and cathode samples in R1; 1821, 2630 and 2082 for sludge, anode and cathode samples in R2. Principal component analysis (PCA) of the six samples was carried out to evaluate the difference of bacterial communities (Fig. 6B). Principal components 1 and 2 explained 54.9% and 31.6% of the total community variations, respectively. Three samples from R2 were clustered together, indicating that microorganisms in sludge significantly affected microbial communities in electrodes. In comparison, the enriched microbial communities at electrodes and sludge were different in R1, especially the anode and sludge samples.

A total of 485 bacterial genera were identified from 6 tested samples. At least 33 genera with relative abundance >1% came from one sample (Table 1). *Enterobacter (*25.29%)*, Enterococcus* (8.83%) and *Desulfovibrio* (5.93%) were abundant in R1-anode. For R2-anode, *Enterococcus* and *Streptophyta* dominated the community with relative abundance of 17.35% and 8.26%, along with *Desulfovibrio* (5.80%). Among these bacteria, *Enterobacter, Enterococcus* and *Desulfovibrio* were reported to be electrochemical active at anode and like implement the anode respiration in R1 and R2^20^,^21^.

Microbial community for R1-cathode was similar to R1-anode, showing *Enterobacter* (16.89%), *Desulfovibrio* (6.26%) and *Gordonibacter* (5.36%). *Enterococcus* genus (21.07%) was predominant at R2-cathode, like R2-anode, but other genera, such as *Desulfovibrio* (10.72%), *Enterobacter* (5.79%) and *Levilinea* (5.48%), were identified. The genera of *Enterobacter, Desulfovibrio* and *Enterococcus* can reduce azo dye or nitro-compounds^1^,^22^. Although this study did not directly ensure that the bio-cathode reduction of AYR was catalyzed by the identified bacteria, the enrichment of these bacteria on cathodes implies that BES creates a niche for the bacteria inhabiting on cathodes. The substantial increase of *Enterobacter* population on electrodes, as compared to sludge indicates that *Enterobacter sp.* plays the key roles of current generation on anodes and AYR reduction on cathodes in R1. In case of electrodes immersing into sludge (R2), *Enterococcus sp.* dominated both of the communities at the sludge and electrodes, which was likely the result of the poor segregation of communities as that was indicated in PCA analysis. The effects of segregation on azo dye decolorization as well as the current generation are still unclear and warrant further study.

## Methods

### Reactor configuration

Three identical cuboid reactors were manufactured with plexiglass, as shown in Fig. 1. Each reactor had a working volume of approximately 1.5 L with the dimensions of L 10 cm × W 5 cm × H 30 cm. A water distribution plate, equispaced with 2 mm holes, was installed at 3 cm over the bottom. Graphite fiber brushes were used for both anodes and cathodes, and each electrode consisted of 8 small graphite fiber brushes (2.5 cm in diameter and 4 cm in length, graphite fiber produced by TOHO TENAX, Co., Ltd., Japan). The distance between anode and cathode in the BES was approximately 2.5 cm. All graphite fiber brushes were pretreated according to the literature^23^. Before assembling the hybrid anaerobic reactors, all the anodes were pre- acclimated in a dual-chamber BES at a fixed anode potential of −400 mV vs. a saturated calomel electrode (SCE) (+247 mV vs. standard hydrogen electrode) controlled by a potentiostat (WMPG1000K8, Wontech International Co., Ltd., Korea). The seed sludge for inoculating the bioanode was the recycled activated sludge obtained from Taiping municipal wastewater treatment plant (Harbin, China) The titanium wire (1 mm in diameter, Baoji LiXing Titanium Group Co., Ltd., China) was coated with heat shrink tube to avoid short circuit and stretched out of the reactors as current collector. During the experiment, each pair of electrodes was operated with external resistance of 10 Ω and applied voltage of 0.5 V using a DC power supply (FDPS-180, Fudan Tianxin Scientific and Educational Instruments Co., Ltd, Shanghai, China). According to our previous work^18^, 0.5 V applied voltage was sufficient for AYR decolorization. Anodes, cathodes and reference electrodes were connected to a data acquisition system (Keithley 2700, Keithley Co. Ltd., US), currents were recorded at every 10 min.

### Inoculation and operation

The anaerobic sludge for R1 and R2 were inoculated with mixture of two sources of sludge in 1:1 ratio:anaerobic sludge from a small pilot-scale anaerobic baffled reactor (ABR)^13^ that had been operated for treating azo dye wastewater for more than 9 months, and anaerobic sludge from a brewery wastewater treatment facility (Harbin, China). The VSS/TSS ratio the sludge was about 0.7. R3 was not inoculated in advance (control test).

The composition of medium was glucose (500 mg/L), AYR (50–200 mg/L), phosphate buffer solution (50 mmol/L), KCl (0.13 g/L), NH_4_Cl (0.31 g/L), trace element solution (10 mL/L) and vitamin solution (10 mL/L)^24^. The influent AYR loading rates (ALR) gradually increased (100, 200, 400, 600, 800 g AYR/m^3^·d) by adjusting the influent AYR concentration and HRT (Table 2). Each operation condition maintained for at least two weeks. The flow rate was controlled with a peristaltic pump (BT100-1 L/YZ1515, Longer Pump Co., Ltd., China). Open and closed circuit modes were compared for the three reactors to assess the contribution of bioelectrochemical effect on AYR removal. All reactors were operated at ambient temperature (23 ± 2 °C).

### Chemicals

AYR that contains an azo bond and a nitro group was used as the model azo dye (Commercial purity grade, Shanghai Sangon Biotech Co., Ltd., China). *p*-phenylenediamine (PPD) and 5-aminosalicylic acid (5-ASA), the main by-products of AYR reduction^18^,^25^, were purchased from Aladdin Industrial Corporation (analytical reagent) and J&K scientific (98.5%), respectively.

### Analytical methods

Liquid samples were immediately filtered through 0.45 μm filters (Tianjin Jinteng Experiment Equipment Co., Ltd., China). AYR concentration was quantified by a UV-Vis spectrophotometer (UV-1800, Shanghai Meipuda instrument Co., Ltd., China) at a wavelength of 374 nm^18^. PPD and 5-ASA were measured by a high performance liquid chromatography (0.3% Acetate: Methanol = 9:1, HPLC, e2695, Waters Co., US) equipped with a UV-Vis detector (288 nm, model 2489, Waters Co., US) and a C18 column (5 μm; 4.6 mm × 150 mm, Symmetry, Waters Co., Ltd., US). Volatile fatty acids (VFAs) was analyzed using a gas chromatograph (GC, 6890 N, Agilent, Inc., US) equipped with a flame ionization detector (FID) and Stabilwax-DA column (30 m × 0.32 mm × 0.5 mm) at oven and injector temperatures of 60 °C and 250 °C, respectively. He carrier gas and N_2_ makeup gas were used for the GC-FID. Chemical oxygen demand (COD) concentration was determined with the dichromate method.

### Calculation

Azo bond cleavage and nitro-reduction decolorize AYR via both anaerobic biological and electrochemical reactions. We built a hypothesis that all electrons transferred to electrodes by anode-respiring microorganisms were used for AYR reduction. Hence, electron recovery efficiency was defined as Eq. (1) to evaluate the portions of AYR decolorization via electrochemical reduction of overall AYR decolorization.


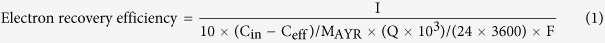


where I is the current which was calculated from the external resistance using Ohm’s law, mA; 10 is electron transfer coefficient, 1 mol AYR reduction requires 10 mol electrons (4 mol for azo bond cleavage and 6 mol for nitro-reduction), mol e^−^/mol AYR; M_AYR_ is AYR’s molecular weight, 287 g/mol; F is Faraday’s constant, 96485 C/mol.

### Biofilms sampling and DNA extraction

Planktonic cells in sludge zone and biofilms on electrodes (anodes and cathodes) in R1 and R2 were sampled for DNA extraction at the end of closed circuit mode operation. Planktonic cells were sampled with a sterile injector from at least three different positions and collected as one sample. Graphite fibers were cut from anodes and cathodes, respectively, and fragmented using sterile scissors. Biofilm samples taken from at least three different small brushes were combined together for DNA extraction. Each sample was put into a 1.5 mL sterile centrifuge tube with 1 mL sterile deionized water, which was then mixed by a shaker flowed by centrifuging at 13 000 rpm for 10 min with removing the supernatants. This procedure was repeated twice for maximizing DNA extraction from biomass samples. Collected pellets were stored in 2 mL sterile centrifuge tubes at −70 °C before DNA extraction. DNA extraction as well as 16S rRNA gene based Illumina MiSeq sequencing of the pellets was served by Shanghai Sangon Biotech Co., Ltd.

### High-throughput 16 S rRNA gene Illumina MiSeq sequencing

Amplicon libraries were constructed by Illumina Miseq 2000 using bacterial universal primers 341 F (5′-CCTACACGACGCTCTTCCGATCTN-3′) and 805 R (5′- GACTGGAGTTCCTTGGCACCCGAGAATTCCA -3′). Both forward and reverse primers were added with barcode. PCR amplification, products purification and quantification, and the sequencing were carried out through the Illumina MiSeq platform in Shanghai Sangon Biotech Co., Ltd. The data analysis was as in Yang *et al*.^26^.

## Additional Information

**How to cite this article**: Cui, M.-H. *et al*. Effect of electrode position on azo dye removal in an up-flow hybrid anaerobic digestion reactor with built-in bioelectrochemical system. *Sci. Rep.* **6**, 25223; doi: 10.1038/srep25223 (2016).

## Figures and Tables

**Table 1:** Relative abundance of bacterial genera in six samples from two reactors.

Genus	R1			R2		
	Sludge	Anode	Cathode	Sludge	Anode	Cathode
Enterococcus	**15.45**	**8.83**	**6.71**	**36.08**	**17.35**	**21.07**
Enterobacter	**1.03**	**25.29**	**16.89**	**6.95**	**3.50**	**5.79**
Desulfovibrio	**6.10**	**5.93**	**6.26**	**3.96**	**5.80**	**10.72**
Levilinea	**5.26**	**2.79**	**4.81**	**2.76**	**3.08**	**5.48**
TM7_genera_incertae_sedis	**5.09**	**1.39**	**1.75**	**6.65**	**2.31**	**4.75**
Alkaliflexus	**3.40**	**1.54**	**2.16**	**3.79**	**1.88**	**4.05**
Gordonibacter	**3.31**	**2.84**	**5.36**	0.75	**4.02**	**2.38**
Bellilinea	**2.78**	**1.79**	**2.54**	**1.43**	**1.83**	**1.08**
Cloacibacillus	**2.62**	**1.96**	**2.09**	0.76	**2.24**	**2.85**
Lactococcus	**2.44**	**4.03**	**4.36**	**3.85**	**1.35**	**2.29**
Solitalea	**2.41**	0.20	**1.43**	**1.89**	0.70	**1.05**
Aminobacterium	**2.27**	0.81	**2.27**	0.39	**2.10**	0.94
Petrimonas	**2.06**	**1.16**	**1.47**	**1.17**	**1.47**	**2.19**
Saccharofermentans	**1.90**	0.44	**1.45**	0.41	**1.49**	0.86
Tissierella	**1.69**	0.12	**1.15**	**1.69**	**1.19**	**1.24**
Anaerovorax	**1.56**	0.39	0.63	0.55	0.98	0.66
Trichococcus	**1.44**	0.76	0.47	**2.92**	**2.80**	**1.04**
Smithella	**1.21**	0.91	**1.85**	0.76	**1.03**	0.30
Geobacter	**1.18**	0.49	**1.02**	0.90	**1.33**	0.43
Prolixibacter	**1.07**	0.18	0.62	0.41	0.89	0.30
Phascolarctobacterium	**1.01**	**1.09**	**1.73**	0.59	0.54	**1.41**
Proteiniphilum	0.99	0.59	0.64	**1.03**	0.68	**1.37**
Dysgonomonas	0.95	0.67	0.33	0.75	0.35	**1.16**
Comamonas	0.90	**1.08**	0.95	0.15	0.58	0.33
Bacillus	0.61	0.04	0.02	0.02	**2.84**	0.01
Klebsiella	0.52	**2.90**	**2.85**	**1.02**	**1.77**	0.78
Anaeroarcus	0.11	**1.77**	**1.10**	0.07	0.11	0.19
Achromobacter	0.10	**1.28**	0.21	0.04	0.11	1.01
Streptophyta	0.09	0.02	0.01	0.01	**8.26**	0.03
Shinella	0.03	**1.57**	0.27	0.02	0.05	0.80
Pannonibacter	0.03	**1.07**	0.38	0.01	0.03	0.06
Sphingopyxis	0.02	**1.42**	0.19	0.02	0.03	0.11
Aquamicrobium	0.02	**1.09**	0.17	0.01	0.01	0.05
Unclassified	**14.63**	**7.44**	**11.16**	**6.92**	**12.75**	**8.75**
Others^a^	**15.73**	**16.13**	**14.71**	**11.29**	**14.57**	**14.48**

Dominant genera (>1%) in the different samples was bolded.

^a^Listed genera were >1% abundances at least one sample, genera with <1% abundances among all six samples were summarized as others.

**Table 2:** Operational conditions at different stages.

Stage	1	2	3	4	5
AYR concentration (mg/L)	50	100	200	200	200
HRT (h)	12	12	12	8	6
AYR loading rate (g-AYR/m^3^·d)	100	200	400	600	800
Glucose concentration (mg/L)	500				
